# Path Coefficient and Principal Component Analyses for Biomass Allocation, Drought Tolerance and Carbon Sequestration Potential in Wheat

**DOI:** 10.3390/plants11111407

**Published:** 2022-05-26

**Authors:** Kwame W. Shamuyarira, Hussein Shimelis, Sandiswa Figlan, Vincent Chaplot

**Affiliations:** 1 School of Agricultural, Earth and Environmental Sciences, University of KwaZulu-Natal, Pietermaritzburg 3201, South Africa; shimelish@ukzn.ac.za (H.S.); vincent.chaplot@ird.fr (V.C.); 2 Department of Agriculture and Animal Health, University of South Africa, Florida 1709, South Africa; figlas@unisa.ac.za; 3 Laboratory of Oceanography and Climate, Experiments and Numerical Approaches (LOCEAN), UMR 7159, IRD/C NRS/UPMC/MNHN, IPSL, 75005 Paris, France

**Keywords:** biomass allocation, drought tolerance, path analysis, principal component analysis, selection efficiency, wheat

## Abstract

Increased root biomass allocation could serve as a proxy trait for selecting crop ideotypes with drought tolerance and carbon sequestration potential in agricultural soils. The objective of this study was to assess the magnitude of the relationship between root biomass and yield components and to identify influential traits so as to optimise genotype selection for enhanced biomass allocation, drought tolerance and carbon sequestration potential in bread wheat (*Triticum aestivum* L.). One-hundred wheat genotypes consisting of 10 parents and 90 derived F_2_ families were evaluated under drought-stressed and non-stressed conditions at two different sites. Data were collected for days to heading (DTH), days to maturity (DTM), plant height, productive tiller number (TN), spike length, spikelets per spike (SPS), kernels per spike (KPS), thousand kernel weight (TKW), shoot biomass, root biomass, total plant biomass (PB), root-to-shoot ratio (RS) and grain yield. There was significant (*p* < 0.05) genetic variation in most assessed traits, TN and RS being exceptions. Root biomass had significant positive correlations with grain yield under drought-stressed (*r* = 0.28) and non-stressed (*r* = 0.41) conditions, but a non-significant correlation was recorded for RS and grain yield. Notably, both root biomass and shoot biomass had significant positive correlations under both water regimes, revealing the potential of increasing both traits with minimal biomass trade-offs. The highest positive direct effects on grain yield were found for KPS and PB under both water regimes. The present study demonstrated that selection based on KPS and PB rather than RS will be more effective in ideotype selection of segregating populations for drought tolerance and carbon sequestration potential.

## 1. Introduction

Soil organic carbon (SOC) is an essential component of soil health, affecting the chemical, physical and biological properties of agricultural soils. In sub-Saharan Africa (SSA), soils of most agricultural lands are degraded due to high soil carbon loss driven by intensive production, poor soil management and crop residue removal [1]. Crop residues present the largest source of carbon (45%) that can be assimilated into the soil through carbon sequestration to improve SOC content [2]. The source of crop residues are root biomass or above-ground plant biomass. However, subsoil additions of carbon from root residues in the form of root litter and root exudates are more efficient in increasing SOC than above-ground plant residues [3]. Furthermore, the residence time in soil of root-derived carbon is 2.4 times that of carbon derived from shoot biomass [4,5]. Hence, soil carbon derived from root biomass is critical for the long-term stabilisation of soil carbon stocks. Promoting below-ground additions of carbon via increased crop root biomass may improve the rate of carbon sequestration in agricultural soils [6].

Breeding wheat (*Triticum aestivum* L., 2n = 6x = 42, AABBDD) with high biomass allocation to roots promises to increase carbon sequestration potential and contribute to yield stability in dryland farming [7]. Crops grown under dryland or rainfed systems are exposed to high fluctuations of water in the soil profile due to rainfall variability and recurrent drought. Plants respond to these fluctuations by altering their biomass allocation patterns [8]. The most unique mechanism by which plants adjust biomass allocation under drought stress is by increasing root-to-shoot ratios, whereby plants allocate more carbohydrates to roots [9]. The accumulation of carbohydrates in the roots stimulates more profound root growth as plants scavenge for moisture to support growth and development [10]. Different studies have shown that root sizes and depths vary significantly within species and between crop cultivars of major cereals, including wheat [11], maize and rice [12]. The genetic variation allows for the identification of contrasting parental genotypes with optimal growth and biomass allocation and agronomic traits to generate new breeding populations for the development of a wheat ideotype with high root biomass and better yield gains.

There is a dearth of information and some debate about whether large root systems impact the increased productivity of wheat in different soil moisture conditions [13,14]. Root biomass has been regarded as an inefficient sink for photo-assimilates, as twice the amount of carbohydrates needed to produce one unit of shoot biomass is needed to produce one unit of root biomass [15]. In high-input crop production systems that use inorganic fertilisers and irrigation, deeper root systems are underestimated, as water and nutrients are readily available [16]. However, recent studies have shown that root system plasticity can be targeted to provide resilience and yield stability under both irrigated and rainfed conditions in arid and semi-arid environments [17,18,19]. High root biomass increases the capacity for and the efficiency of water capture from the soil profile to support plant growth, biomass production, anthesis, grain filling and final grain yield [20]. Ref. [14] reported a 25% increase in shoot biomass production under full irrigation in wheat cultivars with high root biomass relative to those with small root systems. Positive correlations have been observed between seedling root attributes and yield-promoting traits, such as maturity time, kernels per spike, shoot biomass and grain yield, in wheat [21,22]. Therefore, increasing rooting capacity, yield and yield-influencing agronomic traits could stabilise productivity and ensure sustainable grain production of wheat cultivars in different soil moisture conditions [21,23].

The extent of the relationship between root biomass and yield components can be explored through path coefficient and principal component analyses to identify influential traits for cultivar development. In an attempt to develop next-generation wheat cultivars for drought tolerance and carbon sequestration potential, the research group at the University of KwaZulu-Natal identified lines with high genetic variation for desirable traits, such as root biomass, root-to-shoot ratio and grain yield under drought conditions. The selected lines were crossed to produce new breeding populations which were advanced to the F_2_ generation for individual plant selection. Selected plants will be advanced to subsequent generations via pure line or pedigree breeding using complementary technologies, such as doubled haploidy and speed breeding to fast-track selection gains [24,25]. In light of the above background, the objective of this study was to assess the magnitude of the relationship between root biomass and yield components and identify influential traits to optimise selection for enhanced biomass allocation, drought tolerance and carbon sequestration potential in bread wheat. Evaluations of the parental lines and F_2_ families were conducted in field and greenhouse trials. It was hypothesized that there are direct or indirect relationships between and variable contributions made by root biomass and above-ground traits, this hypothesis being used as a guide in the selection of unique genotypes with high root biomass and better grain yield gains.

## 2. Results

### 2.1. Analysis of Variance

The combined analysis of variance revealed significant differences among the test genotypes for the recorded traits, except for TN and RS (Table 1). There were significant differences for RS, TN, DTH, SPS, TKW, spike length and grain yield due to the genotype-by-water regime interaction effect. Significant differences were recorded for DTH, SPS, KPS, TKW, PB, plant height, spike length, shoot biomass, root biomass and grain yield for the genotype-by-site interaction. The genotype-by-water regime-by-site interaction effect was significant only for spike length, SPS and TKW.

### 2.2. Mean Performance

The mean values of measured traits of the ten best performing genotypes and the lowest-performing genotypes based on grain yield under drought-stressed conditions are presented in Table S1. Genotype LM70 × BW141 had the fewest days to heading under drought-stressed and non-stressed conditions. Three of the top four genotypes (LM26 × BW140, BW140 × LM71 and LM47 × BW152) matured later when compared with the lowest-performing genotypes. The top ten genotypes were generally taller than the lowest-performing genotypes, while no clear trends were found for TN for the two groups. Spike length (6.13%) and SPS (5.82%) were reduced slightly by drought stress. Among the lowest-performing genotypes, BW152 (399.15 g·m^−2^) and BW162 × LM71 (334.12 g·m^−2^) had higher shoot biomass under non-stressed conditions than all the top 10 genotypes. However, these values were significantly reduced under drought-stressed conditions. LM75 × LM71 had notably high shoot biomass (>300 g·m^−2^), root biomass (>40 g·m^−2^) and PB (>400 g·m^−2^) values under both moisture conditions but had low yield gain (72.11 g·m^−2^) under drought. The average root-to-shoot ratio for all genotypes increased by 60% due to drought stress. The lowest-performing genotypes had relatively high grain yields (>300 g·m^−2^) under non-stressed conditions. Among the top-performing genotypes, only genotype BW162 × LM75 (436.16 g·m^−2^) had a higher grain yield under non-stressed conditions than all the poor-performing genotypes.

### 2.3. Principal Component and Biplot Analyses

The rotated component matrix showing the percentage variance of different principal components (PCs) and the respective loadings of recorded traits is shown in Table 2. The first four PCs under non-stressed conditions had a cumulative variance of 79.93%. The first PC had the highest variation of 37.04%, followed by PC2 with 15.27%. Shoot biomass, PB and grain yield made the highest contributions to PC1, followed by root biomass, RS and TKW with positive contributions to PC2. The highest positive loadings for PC3 and PC4 were for RS and DTM, respectively. Under drought-stressed conditions, the first PC had a percentage variance of 39.19% and was positively correlated with PB, shoot biomass, SPS, spike length and plant height. The second and third PCs were correlated with DTH and TKW, respectively. Principal components 4 and 5 had high positive loadings for root-related traits, such as RS and root biomass, respectively.

**Figure 1:**
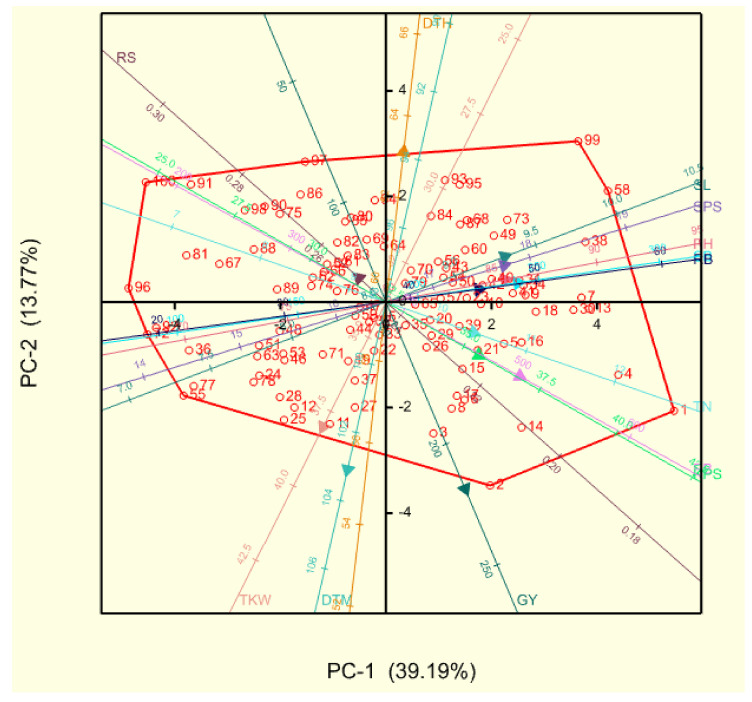
Principal component biplot of 10 bread wheat parental lines and 90 F_2_ families under drought-stressed conditions. Genotypes are coded with numbers, as recorded in Table S1. Smaller angles between vectors of recorded traits indicate a high correlation between the traits in discriminating genotypes. Genotypes plotted closer to and further along a vector line scored highly in that particular trait. PC = principal component, DTH = days to 50% heading, DTM = days to 50% maturity, PH = plant height (cm), TN = tiller number, SL = spike length (cm), SPS = spikelets per spike, KPS = kernels per spike, TKW = thousand kernel weight (g), SB = shoot biomass (g·m^−2^), RB = root biomass (g·m^−2^), PB = total plant biomass (g·m^−2^), RS = root-to-shoot ratio and GY = grain yield (g·m^−2^).

**Figure 2:**
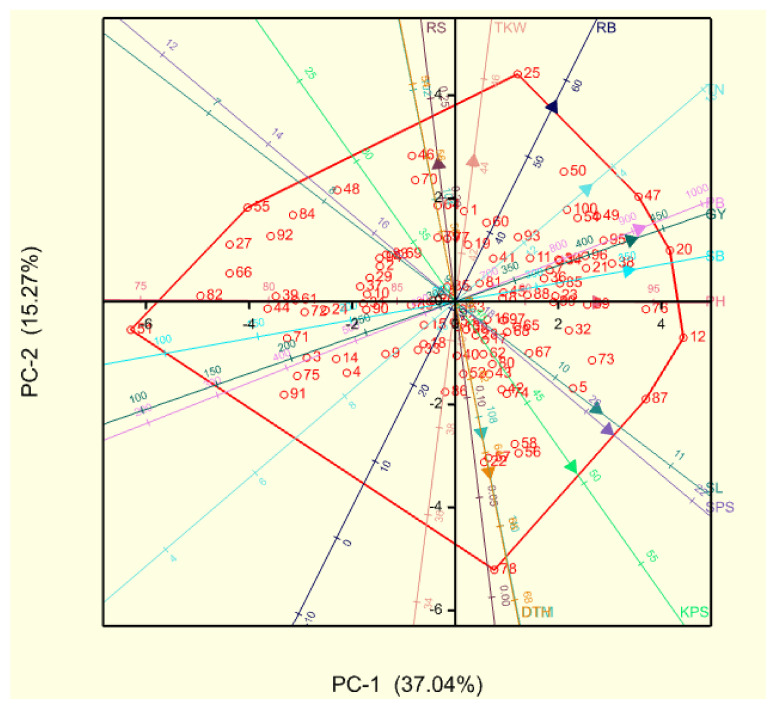
Principal component biplot of 10 bread wheat parental lines and 90 F_2_ families under non-stressed conditions. Genotypes are coded with numbers, as recorded in Table S1. Smaller angles between vectors of recorded traits indicate a high correlation between the traits in discriminating genotypes. Genotypes plotted closer to and further along a vector line scored highly in that particular trait. PC = principal component, DTH = days to 50% heading, DTM = days to 50% maturity, PH = plant height (cm), TN = tiller number, SL = spike length (cm), SPS = spikelets per spike, KPS = kernels per spike, TKW = thousand kernel weight (g), SB = shoot biomass (g·m^−2^), RB = root biomass (g·m^−2^), PB = total plant biomass (g·m^−2^), RS = root-to-shoot ratio and GY = grain yield (g·m^−2^).

Biplots based on the principal component analysis were drawn for drought-stressed and non-stressed conditions (Figure 1 and Figure 2, respectively). The genotypes were evenly scattered across both PC1 and PC2 under drought-stressed conditions. High-yielding genotypes under stress, such as the parental line LM75 and cross BW140 × LM71 had high KPS and PB, while LM26 × BW140 and LM47 × BW152 were also associated with TN (Figure 1 and Table S1). High RS and late flowering were reflected in poor-yielding families, including LM47 × LM71, LM75 × LM71 and BW152 (Figure 1 and Table S1). Under non-stressed conditions, grain yield had strong correlations with PB and TN and high-yielding genotypes under these moisture conditions, including crosses LM71 × LM75 and LM71 × LM26, which excelled in these traits (Figure 2 and Table S1).

### 2.4. Correlations of Root Biomass and Yield Components with Grain Yield under Drought-Stressed and Non-Stressed Conditions

Phenotypic correlation coefficients showing the relationships between root attributes and yield components under drought-stressed and non-stressed conditions are shown in Table 3. Grain yield showed higher positive correlations with PB (*r* = 0.67) and KPS (*r* = 0.55) under drought-stressed conditions. Productive tiller number and shoot biomass had moderate correlations of *r* = 0.33 and *r* = 0.39, respectively, with grain yield, whereas root biomass had low (*r* < 0.30) positive correlations with grain yield. Only DTH had significant negative correlations (*r* = −0.38) with grain yield. Root biomass was positively correlated with RS and all above-ground traits except DTH and DTM. Negative correlations were recorded for RS with plant height (*r* = −0.24), shoot biomass (*r* = −0.31) and PB (*r* = −0.19). 

### 2.5. Path Coefficient Analysis of Root Biomass and Yield Components on Grain Yield

**Figure 3:**
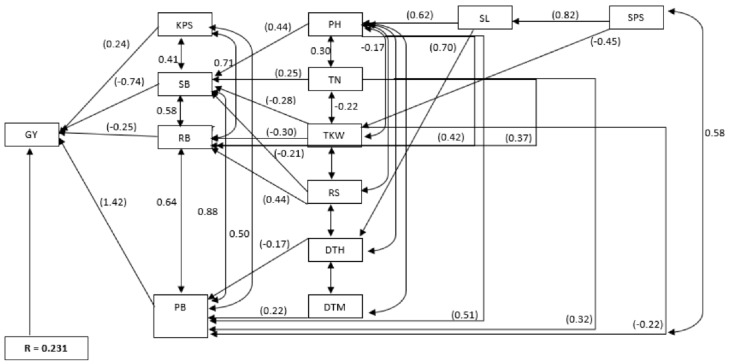
Path analysis model diagram displaying causal relationships of assessed traits on grain yield in 10 bread wheat parental lines and 90 derived F_2_ families of wheat assessed under drought-stressed conditions. Values in parenthesis are direct path coefficients, while other values are correlation coefficients. R = residual effect; DTH = days to 50% heading, DTM = days to 50% maturity, PH = plant height (cm), TN = tiller number, SL = spike length (cm), SPS = spikelets per spike, KPS = kernels per spike, TKW = thousand kernel weight (g), SB = shoot biomass (g·m^−2^), RB = root biomass (g·m^−2^), PB = total plant biomass (g·m^−2^), RS = root-to-shoot ratio and GY = grain yield (g·m^−2^).

**Figure 4:**
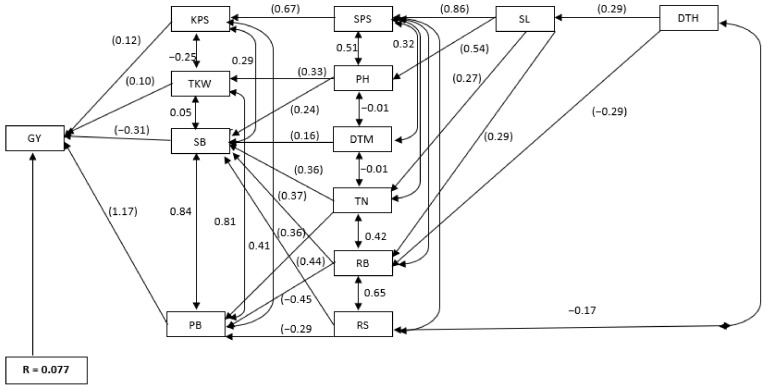
Path analysis model diagram displaying causal relationships of assessed traits on grain yield in 10 bread wheat parental lines and 90 derived F_2_ families of wheat assessed under non-stressed conditions. Values in parenthesis are direct path coefficients, while other values are correlation coefficients. R = residual effect; DTH = days to 50% heading, DTM = days to 50% maturity, PH = plant height (cm), TN = tiller number, SL = spike length (cm), SPS = spikelets per spike, KPS = kernels per spike, TKW = thousand kernel weight (g), SB = shoot biomass (g·m^−2^), RB = root biomass (g·m^−2^), PB = total plant biomass (g·m^−2^), RS = root-to-shoot ratio and GY = grain yield (g·m^−2^).

Direct and indirect effects of yield components and root attributes on grain yield under drought-stressed and non-stressed conditions are summarised in Table S2 and Figure 3 and Table S3 and Figure 4, respectively. PB (1.001) had the greatest direct effect on grain yield under drought-stressed conditions, followed by KPS (0.34) and TKW (0.17). Conversly, shoot biomass and root biomass had negative direct effects on grain yield at −0.410 and −0.153, respectively. Shoot biomass (0.88) and root biomass (0.65) had the greatest positive indirect effects on grain yield through PB. Indirect effects on grain yield through PB were observed for plant height, TN, spike length, SPS and KPS. Root-to-shoot ratio had negative direct effects on grain yield through PB.

Under non-stressed conditions, PB (1.06) and KPS (0.13) had positive direct effects on grain yield. Shoot biomass (−0.19) and root biomass (−0.13) had negative direct effects on grain yield; however, the same traits had large positive indirect effects of 0.89 and 0.52, respectively, on grain yield through PB. Except for RS, assessed traits had positive indirect effects on grain yield through PB, including plant height (0.55), TN (0.66), spike length (0.44), SPS (0.50) and KPS (0.43). The residual value for the path analysis model was 0.077 under drought-stressed conditions and 0.231 under non-stressed conditions (Figure 3 and Figure 4).

## 3. Discussion

Genotypic differences observed for yield components and root biomass under different environments indicate sufficient genetic variation for the development of new wheat ideotypes for grain production and carbon sequestration. The rotated component matrix showed that PB, shoot biomass and grain yield were the most discriminating traits under non-stressed conditions, followed by root biomass, RS and TKW. Therefore, identifying genotypes based on biomass production and allocation will allow more effective explanation of the differences among individual genotypes and families. Similarly, under drought-stressed conditions, biomass production, as shown by high contributions of PB and shoot biomass and spike-related traits, such as SPS and spike length, were the most essential traits in differentiating the test genotypes.

Positive correlations were found between shoot biomass and grain yield under both drought-stressed and non-stressed conditions, indicating the importance of shoot biomass for improving grain yield. High shoot biomass contributes to grain yield by providing greater leaf area for light interception and carbon assimilation to support grain filling [26,27]. This agrees with [28], the authors of which reported that yield gains in wheat are influenced by biomass production and sink size, which is determined by the number of fertile spikes per unit area. To increase the number of fertile spikes per unit area, breeders need to identify plant genotypes with high TN [29]. The importance of TN was notable under drought-stressed conditions, as high yielding genotypes such as LM75 and BW140 × LM71 were strongly correlated with TN. Therefore, increasing shoot biomass and TN will have a positive impact on grain yield.

The positive relationship of root biomass and grain yield shows the importance of root traits for increasing productivity under both drought-stressed and non-stressed conditions. Increase in root size improves the capacity and efficiency of nutrient and moisture acquisition by plants, increasing the resilience of agro-ecosystems. This is more important under drought conditions, where there is limited water in the soil profile and a deeper and more abundant root system can forage for water in the soil profile [30]. Though larger root systems in crops are important, especially in dry areas, they may, in some cases, be inefficient or even entail a yield penalty in wet seasons or in regions with sufficient water and capacity to supply supplementary irrigation [31,32]. However, evidence from this study reflects that root biomass has a positive influence on productivity, even under conditions of sufficient water availability. This is supported by the authors of [33], who reported that deeper and more profuse root growth can be attained without any adverse effect on grain yield. These increased root sizes can be harnessed to increase soil carbon in agricultural soils.

Root-to-shoot ratio was negatively correlated with plant height, shoot biomass and PB under drought, which agrees with [34], whose authors observed negative correlations between root-to-shoot ratio and plant height and above-ground biomass. Some studies debated this and have reported carbon tradeoffs between root and shoot biomass, leading to reduced wheat productivity [35,36,37]. Therefore, increasing RS alone in pursuit of higher carbon inputs may have a negative impact on grain yield, as reflected in families such as LM47 × LM71, LM75 × LM71 and parental line BW152, which had high RS values but low yields. On the other hand, PB had the highest positive correlations with grain yield under both water regimes. Hence, instead of altering RS, yield gains may be obtained by increasing PB without compromising the carbon sequestration potential from the roots. Increasing root biomass and shoot biomass simultaneously to achieve high PB is possible, as reflected by positive correlations between root biomass and shoot biomass under drought-stressed and non-stressed conditions. However, shoot biomass showed stronger indirect effects on grain yield through PB than root biomass under both water regimes. This may indicate the greater contribution to grain yield by shoot biomass than root biomass under different soil moisture environments. Increasing shoot biomass in such cases rather than root biomass may be more beneficial for yield increases but will lead to a considerable reduction in potential carbon inputs in the soil due to reduced root sizes [3,38].

KPS had high correlations with and direct effects on grain yield under both water regimes, corroborating the report of [39]. According to [40], KPS determines the level of grain yield that is achieved in wheat and understanding the fundamental mechanisms that influence KPS will be useful in increasing grain yield. Drought stress reduces KPS due to kernel abortion, which reduces the sink strength in the spikes and causes yield loss [41]. Thus, maintaining a high kernel number, as in the families LM47 × BW152 and LM70 × BW141, under drought may have a positive influence on grain yield. This explains the close relationship between KPS and dry matter accumulation in the spikes, which is reflected in the strong correlations of KPS with spike length and SPS under both drought-stressed and non-stressed conditions [40,42].

The path coefficient analysis models under NS conditions had lower residual values than under drought-stressed conditions. Therefore, the model with non-stressed conditions was more effective in explaining the total variation in grain yield than that with drought-stressed conditions. Both models showed that PB and KPS can be used for efficient selection of grain yield under different soil moisture conditions while pursuing the goal of increasing carbon inputs in croplands. According to [43], SOC levels in the soil are determined by land management practices, such as crop residue retention. Selecting plants with high PB will be beneficial at crop farms that practice minimum tillage and other conservation practices; shoot biomass will provide more crop residues to incorporate into the soil profile after harvest. On the other hand, larger root systems in the same plants will make a high carbon contribution to croplands, especially in areas where conventional agriculture is practised and where there is little contribution from above-ground plant residues.

## 4. Materials and Methods

### 4.1. Plant Material and Population Development

The study used 10 contrasting wheat parental lines selected based on their genetic variation for grain yield and better shoot and root biomass production under drought conditions. The names and pedigrees of the parental genotypes are summarised in Table 4. Eight of the lines were candidate drought- and heat-tolerant lines acquired from the International Maize and Wheat Improvement Center’s (CIMMYT) drought and heat nurseries, and the two lines were local checks adapted to dryland wheat production. The parents were crossed in controlled environment facilities (CEFs) at the University of KwaZulu-Natal (UKZN), South Africa. The parental lines were stagger-planted on three occasions with two-week intervals to allow for synchronised flowering for emasculation and pollination. A total of 90 F1 crosses were developed, including 45 direct crosses and 45 reciprocals using a full diallel mating design. The F1 seeds were harvested and bulked and advanced to the F_2_ generation. The 10 parental lines and their 90 F_2_ families were evaluated under field and greenhouse conditions, as described below.

### 4.2. Phenotyping for Root Biomass and Yield Components

#### 4.2.1. Field Evaluation

- Experimental design and planting

The test genotypes (10 parental lines and 90 F_2_ families) were planted in the field at Ukulinga Research Farm of the University of KwaZulu-Natal (29°40′ S, 30°24′ E; 806 m above sea level). The trial was laid out in a 10 × 10 alpha lattice design with two replications. Genotypes were planted on a 2 m-long plot spaced 30 cm apart, and plants were spaced 20 cm within the row. Three seeds were planted per hole in the experimental unit and thinned to two plants per hill after two weeks of germination. Border rows were used to reduce the risk of yield inflation in test plots in the outer rows. Water and fertiliser were applied through an automated drip irrigation system to ensure that all plants received equal amounts of water.

- Imposing drought stress

The trial was conducted under two water regimes (drought-stressed and non-stressed conditions). Drought-stress was imposed at heading growth stage [44] by withholding irrigation until soil moisture dropped to 35% field capacity, followed by re-watering to 80% field capacity. The non-stressed treatment received water by an automated drip irrigation system to maintain the soil at 80% field capacity. The soil moisture content was monitored by digital moisture sensors (HOBO UX120, Onset, Bourne, MA, USA) inserted to a depth of 60 cm and placed in each replicate of the different water regimes. Standard agronomic practices were used in both water regimes for the duration of the trials according to wheat production guidelines in South Africa [45]. Weather data were recorded for the duration of growth during the trial and are summarised in Table 5.

#### 4.2.2. Greenhouse Evaluation

A greenhouse trial was established at the CEF at the UKZN in Pietermaritzburg, South Africa. The temperature inside the greenhouse was maintained at 25 °C during the day and 15 °C at night. The experimental design followed the field evaluation. Plants were grown in 10 L-capacity plastic pots filled with composted pine bark. Eight seeds were planted in each pot and thinned to five seedlings at two weeks after germination. Water and fertiliser were supplied as described above.

- Imposing drought stress

The trial was conducted under drought-stressed and non-stressed conditions. Irrigation was withheld at the heading stage in the drought-stressed treatment to impose water stress. Rewatering to 80% of field capacity was initiated when moisture dropped to 35% of field capacity to avoid total crop failure. The non-stressed treatment received irrigation four times a day until crop maturity to maintain growing media at 80% of field capacity. Aphids and powdery mildew were chemically controlled using Chess (active ingredient: pyridine azomethine) and Tilt (active ingredient: triazole), respectively. Any emerging weeds were removed manually.

### 4.3. Data Collection

During both field and greenhouse evaluations, data were collected on root biomass, biomass traits and yield components. Days to heading were recorded as the number of days from planting to when 50% of the genotypes in each test plot had fully exposed spikes; days to maturity were recorded as the number of days from planting to when 50% of the genotypes in each test plot had dried spikes; plant height was measured as the height of the plant from the soil to the tip of the spike in centimeters (cm); productive tiller number was recorded as the number of tillers with spikes containing grain at harvest; spike length was the length of the spike, excluding the awns, and expressed in cm; the number of spikelets per spike was counted as the number of spikelets per individual spike; the number of kernels per spike was counted and represented the number of kernels harvested from an individual spike; and thousand kernel weight was taken as the weight of 1000 kernels randomly selected from a genotype and weighed in grams (g). Shoot biomass was recorded as the total above-ground biomass cut from the base of the plant, excluding the grain. The shoots were oven-dried at 70 °C for 48 h, weighed and expressed in g·m^−2^. Root biomass was recorded as the total root dry matter harvested per genotype per plot. Root samples for each plot were harvested to a depth of 50 cm. Large roots were separated from the soil by hand and washed under running water to remove all soil particles. The remaining soil was mixed with water and the suspension was sieved through a 2 mm sieve. Fine roots were collected from the sieve residue and added to the large roots. The roots were oven-dried at 70 °C for 48 h. The dried roots were weighed on a balance to obtain the root biomass, which was adjusted to g·m^−2^. Total plant biomass was the sum of all dry plant material for each genotype, including root biomass, shoot biomass and grain yield, harvested from the test plots and recorded in g·m^−2^. Root-to-shoot ratio was the ratio of the root biomass to the shoot biomass, as recorded above. Grain yield was the weight of harvested grain at 12.5% moisture content per genotype per plot and expressed in g·m^−2^. 

### 4.4. Data Analysis

The data collected across the two sites were individually analysed and subjected to Bartlet’s homogeneity test of variance. A combined analysis of variance (ANOVA) was computed to test genotype effects using a lattice procedure involving a three-way interaction of genotype, water regime and site, using Genstat, 18th edition [46]. Mean values of the test genotypes for the assessed traits were compared at the 5% significance level using Fisher’s least significance difference (LSD) procedure. Pearson’s correlation coefficients (*r*) were calculated separately for drought-stressed and non-stressed conditions using Genstat, 18th edition [46]. Correlation coefficients were partitioned into direct and indirect effects using the phenotypic correlation matrix: A = B × C, where A is the phenotypic correlation coefficients of grain yield with yield components and root attributes, B is the phenotypic correlations among all the recorded traits in all combinations and C is the value of the path coefficients. The inverse of matrix B was calculated in Microsoft Excel 2016, using the Matrix Inverse function (MINVERSE). The direct and indirect path coefficients of yield components and root biomass were calculated as the products of phenotypic correlations and inverse matrix B, according to [47]. Path coefficient analysis diagrams were developed in R software using structural equation modelling [48]. The rotated component matrix and principal component analysis (PCA) biplots were generated for yield components and root biomass based on the correlation matrix in Genstat, 18th edition. Both analyses were separated for drought-stressed and non-stressed conditions.

## 5. Conclusions

Increasing RS was not effective with respect to improving wheat productivity. The positive relationship between root biomass and shoot biomass informs the possibility of increasing both traits with minimum biomass trade-offs to develop drought-tolerant cultivars with high carbon sequestration potential. The large positive direct effects of KPS and PB on grain yield under both water regimes point to plants with high root and shoot biomass production as the crop ideotype that will be most resilient and environmentally friendly under varying moisture conditions. This was observed for high-yielding genotypes, including LM75 and BW140 × LM71 under drought-stress and LM71 × LM75 and LM71 × LM26 under non-stressed conditions. Therefore, selection based on KPS and PB rather than RS will be more effective in ideotype selection of segregating F_2_ populations with enhanced drought tolerance and carbon sequestration potential. Selected plants and families will be advanced using pedigree breeding to develop climate-smart cultivars for dryland farming in SSA. The study recommends further evaluations across multiple environments and test populations to develop genetic models and to guide the breeding of climate-smart wheat varieties with drought tolerance and high carbon sequestration potential.

## Figures and Tables

**Table 1:** Combined analysis of variance and significance tests for yield components and root attributes of 10 parental lines and their 90 F_2_ progenies evaluated under drought-stressed and non-stressed conditions across two sites.

SOV	d.f	DTH	DTM	PH	TN	SL	SPS	KPS	TKW	SB	RB	PB	RS	GY
Replication	1	30.06	228.44 ***	142.45	335.34 ***	5.95 *	18.01 *	212.15	421.16 ***	4484.00	10,390.90 ***	220,237.00 *	0.49 ***	43,869.00 *
Block	18	29.41 ***	27.11	176.89 *	21.30	2.06 *	6.352	115.63 *	56.25 **	25,982.00 ***	994.50 *	67,304.00	0.04	21,502.00 **
Genotype (Gen)	99	60.16 ***	41.60 ***	243.27 ***	16.10	4.09 ***	11.61 ***	142.25 ***	58.08 ***	18,128.00 ***	769.50 **	69,654.00 ***	0.02	14,638.00 **
Water Regime (WR)	1	36.23	11,794.04 ***	11,091.44 ***	205.42 **	59.49 ***	202.55 ***	7189.67 ***	9737.33 ***	938,657.00 ***	10,709.50 ***	12,251,041.00 ***	1.68 ***	5,465,271.00 ***
Site	1	46,292.11 ***	128,227.14 ***	89,648.69 ***	15,460.90 ***	1630.17 ***	5094.70 ***	67,179.66 ***	64,551.23 ***	2,054,036.00 ***	24,588.50 ***	1,132,252.00 ***	0.05	165,356.00 ***
Gen * WR	99	13.34 *	20.84	104.61	20.34 *	1.59 *	7.20 ***	71.70	32.42 *	11,642.00	631.90	53,980.00	0.03 *	13,481.00 *
Gen * Site	98	37.36 ***	17.86	149.27 ***	17.57	2.28 ***	10.32 ***	94.62 *	32.11 *	17,491.00 ***	756.10 *	67,155.00 **	0.03	14,496.00 *
Gen.WR.Site	97	12.37	17.73	116.64	13.40	1.73 **	7.13 ***	72.21	37.09 ***	12,166.00	577.70	45,619.00	0.03	12,154.00
Residual	368	9.79	19.02	91.79	14.67	1.148	4.352	66.81	23.58	10,071.00	533.00	43,026.00	0.03	10,167.00

* Significant at *p* < 0.05, ** *p* < 0.01, *** *p* < 0.001, SOV = source of variation, df = degrees of freedom, DTH = days to 50% heading, DTM = days to 50% maturity, PH = plant height (cm), TN = tiller number, SL = spike length (cm), SPS = spikelets per spike, KPS = kernels per spike, TKW = thousand kernel weight (g), SB = shoot biomass (g·m^−2^), RB = root biomass (g·m^−2^), PB = total plant biomass (g·m^−2^), RS = root-to-shoot ratio and GY = grain yield (g·m^−2^).

**Table 2:** Rotated component matrix for 13 yield components and root attributes in 100 wheat genotypes assessed under drought-stressed and non-stressed conditions.

Traits	Non-Stress				Drought-Stress				
	PC1	PC2	PC3	PC4	PC1	PC2	PC3	PC4	PC5
DTH	0.08	−0.38	0.17	−0.33	0.05	0.44	0.35	0.22	0.33
DTM	0.06	−0.29	0.01	0.45	−0.11	−0.49	0.18	0.34	0.27
PH	0.32	0.00	0.25	−0.29	0.35	0.07	0.33	0.07	0.10
TN	0.29	0.25	−0.27	0.04	0.26	−0.09	−0.30	−0.25	0.26
SL	0.32	−0.24	0.36	−0.12	0.35	0.13	0.26	0.24	−0.16
SPS	0.34	−0.29	0.24	0.07	0.35	0.11	0.14	0.25	−0.21
KPS	0.26	−0.37	0.03	0.22	0.27	−0.16	0.00	0.14	−0.60
TKW	0.04	0.31	0.24	−0.56	−0.18	−0.37	0.42	0.20	0.25
SB	0.39	0.07	−0.28	−0.05	0.39	0.06	0.05	−0.20	0.23
RB	0.22	0.43	0.28	0.28	0.27	0.04	−0.43	0.25	0.39
PB	0.41	0.16	−0.21	0.04	0.39	−0.22	−0.06	−0.09	0.19
RS	−0.04	0.31	0.60	0.37	−0.08	0.07	−0.44	0.69	−0.03
GY	0.39	0.14	−0.19	0.05	0.23	−0.55	−0.05	−0.02	−0.10
Eigenvalue	4.82	1.99	1.40	1.35	5.10	1.79	1.52	1.23	1.02
Per Var (%)	37.04	15.27	10.74	10.35	39.19	13.77	11.66	9.45	7.81
Cum Var (%)	37.04	52.31	63.05	73.40	39.19	52.96	64.62	74.07	81.88

PC = principal component, Per Var = percentage variance, Cum Var = cumulative variance, DTH = days to 50% heading, DTM = days to 50% maturity, PH = plant height, TN = tiller number, SL = spike length, SPS = spikelets per spike, KPS = kernels per spike, TKW = thousand kernel weight, SB = shoot biomass, RB = root biomass, PB = total plant biomass, RS = root-to-shoot ratio and GY = grain yield.

**Table 3:** Pearson’s correlation coefficients and significance tests for yield components and root attributes of 10 bread wheat parental lines and 90 F_2_ progenies under drought-stressed (lower diagonal values) and non-stressed (upper diagonal values) conditions at two sites.

Traits	DTH	DTM	PH	TN	SL	SPS	KPS	TKW	SB	RB	PB	RS	GY
DTH	1	0.03	0.23 *	−0.05	0.21 *	0.13	0.12	0.02	0.1	−0.16	0.03	−0.17	0.02
DTM	−0.29 **	1	−0.01	−0.01	0.05	0.32 **	0.05	−0.21 *	0.11	0.03	0.03	−0.01	0.02
PH	0.28 **	−0.25 *	1	0.32 **	0.55 ***	0.51 ***	0.28 **	0.32 **	0.53 ***	0.31 **	0.51 ***	−0.05	0.48 ***
TN	−0.04	−0.1	0.30 **	1	0.22 *	0.24 *	0.15	0.05	0.59 ***	0.42 ***	0.62 ***	0.03	0.60 ***
SL	0.27 **	−0.27 **	0.74 ***	0.31 **	1	0.76 ***	0.49 ***	0.1	0.45 ***	0.22 *	0.41 ***	−0.06	0.41 ***
SPS	0.13	−0.15	0.64 ***	0.32 **	0.79 ***	1	0.62 ***	−0.04	0.49 ***	0.2	0.47 ***	−0.07	0.48 ***
KPS	−0.04	−0.06	0.39 ***	0.25 *	0.49 ***	0.55 ***	1	−0.25 *	0.29 **	0.04	0.40 ***	−0.11	0.48 ***
TKW	−0.13	0.37 ***	−0.17 **	−0.22 *	−0.24 *	−0.38 ***	−0.29 **	1	0.05	0.09	0.1	0.08	0.13
SB	0.15	−0.23 *	0.71 ***	0.55 ***	0.63 ***	0.59 ***	0.41 ***	−0.40 ***	1	0.37 ***	0.84 ***	−0.24 *	0.72 ***
RB	0.06	−0.03	0.37 ***	0.51 ***	0.36 ***	0.40 ***	0.23 *	−0.33 ***	0.58 ***	1	0.49 ***	0.65 ***	0.41 ***
PB	−0.06	−0.03	0.60 ***	0.57 ***	0.54 ***	0.58 ***	0.50 ***	−0.26 **	0.88 ***	0.64 ***	1	−0.05	0.94 ***
RS	0.03	0.1	−0.24 *	−0.06	−0.13	−0.13	−0.11	−0.05	−0.31 **	0.39 ***	−0.19 ***	1	−0.03
GY	−0.38 ***	0.24 *	0.26 **	0.33 ***	0.24 *	0.25 *	0.55 ***	0.09	0.39 ***	0.28 **	0.67 ***	−0.14	1

* Significant at *p* < 0.05, ** *p* < 0.01, *** *p* < 0.001, DTH = days to 50% heading, DTM = days to 50% maturity, PH = plant height (cm), TN = tiller number, SL = spike length (cm), SPS = spikelets per spike, KPS = kernels per spike, TKW = thousand kernel weight (g), SB = shoot biomass (^−2^), RB = root biomass (g·m^−2^), PB = total plant biomass (g·m^−2^), RS = root-to-shoot ratio and GY = grain yield (g·m^−2^). The majority of the traits had significant positive correlations with grain yield under non-stressed conditions. Highly significant correlations were recorded between grain yield and PB (r = 0.94) and between shoot biomass (*r* = 0.72) and TN (0.60). Plant height (*r* = 0.48), SPS (*r* = 0.48), KPS (*r* = 0.48), root biomass (*r* = 0.41) and spike length (*r* = 0.41) showed moderate correlations with grain yield. Productive tiller number and SL had positive correlations with shoot biomass, PB and root biomass. However, spike characteristics, including SPS and KPS, were positively correlated with shoot biomass and PB but had non-significant correlations with root biomass. Root-to-shoot ratio had significant correlations with root biomass (*r* = 0.65) and shoot biomass (*r* = −0.24). Positive correlations were recorded for root biomass with PH, TN, spike length, shoot biomass and PB. Notably, under both water regimes, RS had non-significant and negative correlations with grain yield.

**Table 4:** List of the parental lines used in the crosses.

Genotype	Pedigree
LM26	ATTILA * 2/PBW65//TAM200/TUI
LM47	FRET2/KUKUNA//FRET2/3/YANAC/4/FRET2/KIRITATI
LM48	FRET2/KUKUNA//FRET2/3/PASTOR//HXL7573/2 * BAU/5/FRET2 *2/4/SNI/TRAP#1/3/KAUZ * 2/TRAP//KAUZ
LM71	BABAX/3/PRL/SARA//TSI/VEE#5/4/CROC_1/AE.SQUARROSA (224)//2 * OPATA
LM75	BUC/MN72253//PASTOR
BW141	CGSS05B00243T-099TOPY-099M-099NJ-099NJ-1WGY-0B
BW152	CGSS05B00258T-099TOPY-099M-099NJ-1WGY-0B
BW162	CGSS05B00304T-099TOPY-099M-099NJ-099NJ-3WGY-0B
LM70	Local check
BW140	Local check

* backcross.

**Table 5:** Monthly weather data during the field trial at Ukulinga Research Farm, Pietermaritzburg, South Africa (2020).

Month	Max Temp (°C)	Min Temp (°C)	Humidity (%)	Rain (mm)
July	19.9	5.4	60	31
August	21.8	7.7	60	37
September	23.1	10	67	59
October	23.3	12	75	100
November	23.7	13.5	79	121
December	24.8	15.3	81	137

Max Temp = average maximum temperature, Min Temp = average minimum temperature.

## Supplementary Materials

The following supporting information can be downloaded at: https://www.mdpi.com/article/10.3390/plants11111407/s1, Table S1: Mean values of yield components and root attributes of the ten parental lines and 90 F_2_ families of wheat based on grain yield under drought-stressed conditions. Table S2. Direct (bold face values) and indirect effects for yield components and root attributes on grain yield of 10 bread wheat parental lines and 90 F_2_ progenies under drought-stressed conditions at two sites. Table S3. Direct (bold face values) and indirect effects for yield components and root attributes on grain yield of 10 bread wheat parental lines and 90 F_2_ progenies under non-stressed conditions at two sites.
