# Supplementary material for: Path Coefficient and Principal Component Analyses for Biomass Allocation, Drought Tolerance and Carbon Sequestration Potential in Wheat
Source: Plants (Basel). 2022 May 26;11(11):1407. doi: 10.3390/plants11111407 (PMC9182935; doi:10.3390/plants11111407)
Supplement: Supplementary file 1 [file plants-11-01407-s001.zip › plants-1741000-supplementary.pdf]

Supplementary Tables

Table S1 Mean values of yield components and root attributes of the ten parental lines and 90 F_2 families of wheat based on grain yield under drought-stressed conditions.

No.	Genotype	DTH		DTM		PH		TN		SL		SPS		KPS		TKW	
		NS	DS	NS	DS	NS	DS	NS	DS	NS	DS	NS	DS	NS	DS	NS	DS
**1**	LM75	60.75	61.25	102.00	97.75	92.58	89.03	9.56	12.56	8.64	9.08	17.50	17.50	35.54	39.96	40.53	30.64
**2**	LM26 x BW140	53.75	59.75	103.75	106.00	80.88	75.20	10.78	13.31	8.80	9.10	16.63	16.00	37.33	36.00	36.90	34.20
**3**	BW140 x LM71	55.25	57.00	103.50	99.25	78.75	76.48	8.00	9.81	8.58	8.61	16.75	16.63	41.38	39.75	37.25	35.89
**4**	LM47 x BW152	59.25	59.50	107.00	100.00	83.30	92.13	10.19	10.13	9.48	9.38	16.88	17.63	35.79	40.88	39.66	30.35
**5**	LM70 x BW152	65.25	60.50	106.25	98.25	95.45	85.00	14.25	10.31	9.95	9.75	19.13	18.13	42.63	32.25	39.97	34.45
**6**	LM70 x BW141	56.50	54.50	106.50	95.00	90.45	83.73	7.63	10.38	9.85	8.67	18.38	17.25	45.21	40.21	42.48	36.82
**7**	BW162 x LM75	60.50	63.00	102.50	96.75	94.85	92.03	10.88	9.44	9.48	9.18	17.63	18.00	39.67	38.25	41.83	31.88
**8**	LM75 x BW141	59.00	54.50	104.25	98.25	86.23	79.55	11.88	12.31	9.56	8.58	19.38	17.38	41.96	34.29	42.45	35.37
**9**	BW152 x BW141	62.25	59.75	105.50	94.25	86.55	84.90	6.83	9.38	9.55	8.99	17.88	16.63	41.83	32.83	43.83	32.26
**10**	LM70 x BW162	62.50	60.25	103.50	96.00	84.58	78.58	9.94	12.13	8.93	9.45	16.88	18.00	36.33	33.88	43.85	33.33
**11**	BW141 x LM26	55.25	56.75	108.25	104.25	89.43	75.93	11.92	8.31	10.18	8.25	18.50	16.00	36.54	37.71	43.92	35.99
**12**	LM48 x BW140	57.50	58.00	111.50	103.25	85.58	72.13	12.94	6.81	10.13	7.43	21.63	14.63	48.42	33.88	37.21	32.84
**13**	LM75 x LM47	60.25	59.75	105.75	94.75	95.75	91.15	7.13	13.06	9.80	10.00	18.25	17.50	37.25	36.00	44.99	33.77
**14**	LM70 x LM26	55.75	58.33	106.00	105.00	85.60	83.67	7.38	9.33	9.59	9.88	17.25	18.50	34.96	40.17	39.51	39.38
**15**	LM47 x BW140	61.50	58.25	108.25	99.75	86.55	79.30	11.38	11.25	9.05	8.75	17.75	16.50	34.08	34.67	41.43	34.35
**16**	LM26 x LM71	60.75	57.00	107.75	100.00	93.13	87.20	9.88	10.75	9.39	9.43	17.75	18.00	36.75	34.83	36.35	29.67
**17**	BW141 x LM75	59.25	55.25	106.75	100.50	95.43	87.18	11.56	10.50	8.55	9.00	18.13	17.13	43.54	31.79	41.58	37.10
**18**	BW162 x LM26	59.25	55.50	108.00	95.25	83.45	84.83	9.31	12.44	9.08	9.19	18.63	19.00	42.38	34.25	38.49	31.47
**19**	LM26 x BW141	54.50	56.00	105.75	99.50	90.40	71.65	11.31	6.44	8.58	8.63	17.75	16.88	41.75	38.79	40.67	31.24
**20**	LM71 x LM26	55.75	56.00	110.25	98.00	88.70	78.38	13.63	7.63	10.50	8.83	20.25	17.50	35.21	31.21	40.26	29.62
**21**	BW152 x LM75	59.75	58.25	107.25	99.00	89.28	83.60	12.41	11.63	9.70	9.00	19.00	18.75	40.67	35.46	43.69	35.41
**22**	LM48 x BW162	69.50	61.67	112.00	101.33	98.80	84.33	6.50	7.67	9.57	8.16	19.00	16.38	38.67	36.50	45.00	37.29
**23**	LM75 x BW152	60.75	63.00	105.00	96.50	93.33	82.68	12.56	10.75	9.40	9.45	18.75	17.38	41.92	37.75	42.25	37.63
**24**	BW152 x LM26	56.25	57.50	106.75	99.75	81.58	75.48	9.31	7.75	8.38	7.15	17.75	15.00	30.33	30.17	36.58	36.86
**25**	BW140 x LM75	55.75	56.25	105.25	100.75	85.55	71.70	13.94	9.94	8.00	7.60	15.75	14.38	31.63	28.96	45.75	40.19
**26**	BW140 x BW152	58.75	57.50	109.50	98.75	85.00	80.60	11.81	13.94	8.50	7.88	18.25	16.63	36.42	28.29	43.45	34.48
**27**	BW162 x BW140	55.25	56.00	103.00	102.50	75.90	73.18	9.31	12.50	6.70	7.83	12.88	14.88	33.92	30.17	37.81	34.71
**28**	LM75 x BW140	56.50	56.75	112.00	100.50	86.10	78.30	9.13	9.31	8.98	6.33	19.38	13.38	38.92	25.92	40.09	36.42
**29**	LM71 x BW140	57.25	55.50	109.25	98.25	75.78	75.58	8.75	8.81	7.38	9.56	14.25	17.63	38.08	34.46	36.08	31.32
**30**	BW141 x LM70	58.75	60.75	103.75	96.25	96.38	91.18	12.38	11.31	9.46	9.24	17.13	17.75	38.83	34.63	46.73	35.31

DTH = days to 50% heading, DTM = days to 50% maturity, PH = plant height (cm), TN = tiller number, SL = spike length (cm), SPS = spikelets per spike, KPS = kernels per spike, TKW = thousand kernel weight (g), NS = non-stressed, DS = drought-stressed

Table S1 continued.

No.	Genotype	DTH		DTM		PH		TN		SL		SPS		KPS		TKW	
		NS	DS	NS	DS	NS	DS	NS	DS	NS	DS	NS	DS	NS	DS	NS	DS
**31**	LM75 x LM48	62.25	61.50	103.75	98.00	83.30	86.33	8.94	12.19	8.27	9.13	15.50	17.25	36.08	35.96	40.15	29.25
**32**	BW141 x BW152	61.50	59.75	111.00	100.25	99.40	82.03	12.50	7.19	9.57	8.00	18.67	16.50	38.63	33.43	39.06	31.39
**33**	LM75 x LM70	62.75	58.25	106.25	96.25	91.50	88.45	9.06	7.13	9.38	8.95	17.50	16.38	38.46	34.79	40.84	39.54
**34**	LM70 x LM47	59.25	64.25	101.50	98.75	96.28	92.75	11.94	10.06	9.63	9.50	17.88	16.75	43.67	34.25	48.34	34.76
**35**	LM26 x LM48	57.00	57.25	104.50	98.00	85.08	79.63	11.06	7.00	8.68	9.00	15.88	17.63	44.08	45.58	36.73	31.80
**36**	BW162	64.75	59.25	102.50	100.75	93.13	70.05	13.56	6.00	9.13	7.13	17.88	13.75	38.88	23.67	44.76	36.34
**37**	BW140 x LM70	55.25	55.50	105.00	102.00	79.50	80.60	8.38	8.50	8.80	8.95	16.50	17.25	39.46	33.92	42.24	39.23
**38**	LM70 x LM75	62.50	62.00	107.00	93.00	97.43	94.90	8.75	11.69	9.60	9.90	17.88	17.75	38.08	41.33	44.46	31.01
**39**	LM47 x LM70	63.75	63.75	104.50	102.50	85.93	85.78	8.75	11.69	8.05	8.89	14.63	16.38	28.33	31.08	40.11	34.72
**40**	LM47 x LM48	62.00	56.25	101.25	94.50	89.30	88.68	11.19	7.88	9.85	8.95	17.38	17.38	43.17	35.25	35.35	27.73
**41**	LM26 x BW162	55.25	59.50	105.25	97.50	88.08	78.15	11.56	10.00	9.00	9.45	16.63	18.00	43.75	34.17	40.40	31.43
**42**	LM48 x LM26	62.75	61.25	107.25	97.75	82.05	76.75	12.06	10.44	9.43	8.80	18.25	17.88	51.25	37.96	37.84	30.42
**43**	BW162 x LM48	60.75	59.25	101.50	94.00	85.03	80.43	9.06	8.88	10.14	8.75	18.75	15.88	47.75	35.54	34.12	31.78
**44**	BW141	55.50	57.25	104.75	96.75	74.60	74.95	9.88	11.31	7.88	7.69	16.13	16.63	35.63	34.04	38.94	33.60
**45**	LM75 x BW162	63.50	57.50	106.50	95.50	89.40	81.23	12.19	13.25	9.38	8.03	18.13	15.25	34.38	30.75	44.02	34.33
**46**	BW140 x BW162	51.33	53.75	104.67	96.75	84.70	74.30	13.43	7.75	7.77	8.28	15.83	16.13	32.89	36.08	40.60	37.15
**47**	LM71 x LM75	59.75	60.75	104.25	98.00	86.15	82.08	15.75	10.19	9.15	8.30	17.63	14.75	39.29	30.79	41.01	35.63
**48**	LM70 x BW140	58.75	58.25	107.75	97.50	86.00	81.80	8.19	6.81	9.13	8.45	17.13	15.25	33.42	26.46	43.10	40.40
**49**	LM26 x LM75	62.50	60.50	104.25	94.25	91.40	86.95	13.81	12.13	8.83	9.13	17.00	16.63	37.42	33.54	42.29	28.99
**50**	BW162 x BW141	55.25	59.00	105.75	98.25	95.23	84.93	9.16	10.38	9.88	9.05	18.63	18.75	38.88	32.71	44.88	32.01
**51**	BW140 x LM48	58.25	58.50	107.50	98.25	76.40	73.25	6.13	9.19	6.63	7.38	13.13	15.00	28.75	34.04	37.84	37.82
**52**	LM26	59.25	56.75	103.50	95.50	83.90	69.05	10.19	7.81	9.48	8.68	17.63	17.38	44.58	41.63	35.01	29.34
**53**	LM48 x BW141	60.75	59.00	111.75	102.50	88.63	70.08	10.56	9.56	9.23	7.68	17.75	14.63	36.38	34.46	38.41	32.13
**54**	LM47 x LM26	58.25	58.75	109.25	97.75	90.48	86.30	11.75	9.31	9.33	9.15	18.25	18.00	41.11	34.63	49.50	30.85
**55**	BW140 x BW141	53.00	56.25	106.00	101.75	74.90	61.03	10.31	10.81	6.88	7.00	14.38	13.50	30.71	29.75	45.38	36.47
**56**	BW152 x LM48	66.75	61.00	107.50	97.50	91.00	81.90	9.69	9.13	8.95	8.75	19.25	17.75	50.33	38.63	33.56	29.56
**57**	BW140	63.67	57.00	118.00	99.00	79.20	78.55	9.81	11.44	8.93	8.64	18.25	18.75	48.92	38.13	34.96	28.95
**58**	LM70 x LM71	62.25	61.00	108.25	92.25	81.88	89.70	11.31	10.31	11.88	10.19	22.63	19.00	45.06	35.08	41.67	29.27
**59**	LM26 x LM47	59.25	56.00	107.25	97.75	93.15	85.23	12.94	7.00	10.43	8.68	18.88	16.63	42.63	31.46	42.87	36.44
**60**	BW162 x BW152	60.00	62.50	100.25	97.75	90.60	80.48	11.00	9.81	9.56	9.50	18.13	18.13	37.92	31.04	41.82	32.61

DTH = days to 50% heading, DTM = days to 50% maturity, PH = plant height (cm), TN = tiller number, SL = spike length (cm), SPS = spikelets per spike, KPS = kernels per spike, TKW = thousand kernel weight (g), NS = non-stressed, DS = drought-stressed

Table S1 continued.

No.	Genotype	DTH		DTM		PH		TN		SL		SPS		KPS		TKW	
		NS	DS	NS	DS	NS	DS	NS	DS	NS	DS	NS	DS	NS	DS	NS	DS
**61**	LM71 x BW152	62.67	62.25	108.00	96.50	76.78	76.75	7.06	11.63	7.88	7.78	14.75	15.50	29.75	28.92	42.76	33.02
**62**	LM48 x LM47	64.25	60.50	104.50	96.50	96.33	75.65	11.38	12.56	10.00	8.69	18.13	15.50	36.83	28.38	40.00	36.33
**63**	BW140 x LM26	56.75	56.00	110.75	101.75	85.83	78.45	13.00	5.36	8.48	7.81	18.00	15.63	35.54	28.88	34.79	36.18
**64**	LM47 x LM75	57.67	61.00	100.00	95.75	87.90	82.05	11.33	8.75	9.00	8.91	17.17	16.38	33.56	26.08	47.41	34.13
**65**	LM26 x LM70	56.75	57.00	105.00	98.75	92.78	82.68	9.63	9.00	10.53	9.54	19.44	17.50	35.89	34.67	39.96	32.78
**66**	LM71 x LM70	62.00	58.25	104.00	95.75	74.63	78.40	6.75	7.69	8.30	9.00	14.00	16.13	28.54	25.83	46.39	36.87
**67**	LM48 x LM75	60.75	61.00	102.50	96.75	89.35	69.80	9.44	6.38	9.50	7.70	19.00	14.38	46.50	32.88	36.79	33.69
**68**	LM48 x LM70	61.50	63.25	104.00	96.25	88.75	85.33	7.75	10.38	9.73	9.00	17.50	17.38	39.50	37.21	43.72	30.69
**69**	LM48 x BW152	62.25	60.75	102.75	96.50	83.80	87.43	10.50	7.55	9.18	8.70	15.75	15.75	39.88	29.21	38.67	32.42
**70**	BW140 x LM47	61.00	60.25	108.75	97.25	91.88	79.45	13.75	9.81	8.65	8.20	15.38	16.25	32.96	26.88	41.38	34.61
**71**	LM71 x BW141	57.50	56.25	110.50	100.50	74.00	77.18	7.06	11.60	8.50	7.75	15.88	15.38	36.08	30.50	33.88	36.31
**72**	BW141 x LM71	56.75	54.75	107.00	96.75	84.18	65.05	7.81	9.13	8.53	6.70	17.13	13.25	31.04	26.83	41.82	35.33
**73**	LM70	64.00	62.25	105.00	95.50	91.08	84.98	11.06	9.81	10.25	9.74	18.75	18.38	44.21	36.25	43.99	32.07
**74**	BW152 x BW140	58.75	54.75	112.00	98.00	86.08	74.33	9.25	9.81	8.68	7.60	19.63	15.83	46.38	20.39	38.87	28.88
**75**	BW152 x BW162	63.33	64.00	105.25	96.75	80.85	73.40	6.63	8.00	7.98	7.95	16.38	17.38	38.17	28.13	38.56	32.80
**76**	LM26 x BW152	60.00	62.33	107.00	101.00	91.10	80.73	12.31	8.75	9.87	8.73	20.00	17.25	48.54	30.29	37.27	37.82
**77**	BW141 x LM48	55.00	61.00	104.00	104.33	84.13	72.60	15.69	7.50	8.08	7.53	15.88	14.88	41.50	32.44	39.37	44.97
**78**	LM70 x LM48	70.00	66.00	112.50	112.00	93.90	91.30	8.25	4.25	10.60	9.30	20.00	18.00	53.67	23.67	37.50	50.00
**79**	LM47 x BW141	56.00	56.00	102.75	99.25	98.75	79.88	11.13	8.94	8.51	8.81	16.38	18.13	37.00	27.75	43.16	33.01
**80**	LM71 x LM47	61.50	63.25	103.25	96.25	88.93	83.43	10.50	7.46	10.93	9.35	18.63	16.50	45.88	33.75	43.48	36.21
**81**	BW141 x LM47	58.25	61.25	108.00	97.00	98.40	71.38	11.44	4.63	9.73	8.03	18.38	14.38	33.00	28.63	43.22	37.56
**82**	BW162 x LM70	64.67	61.50	104.00	96.75	72.35	82.08	8.00	9.81	8.56	9.58	15.38	17.00	25.00	31.79	50.00	38.58
**83**	BW162 x LM47	64.50	60.50	107.00	96.25	84.50	81.45	12.94	9.25	7.63	8.03	14.63	15.25	35.42	28.17	39.60	34.66
**84**	BW152 x LM71	59.50	61.75	103.25	96.50	83.95	83.50	5.19	9.88	7.98	9.24	15.00	17.13	31.58	34.71	45.34	30.91
**85**	LM71 x BW162	63.00	61.00	105.00	97.00	88.75	78.70	14.06	9.56	9.20	9.40	18.13	16.25	35.75	29.75	39.93	32.92
**86**	BW152 x LM47	63.75	64.75	106.50	95.75	89.73	77.03	8.50	8.06	9.20	7.75	18.50	15.88	41.38	29.79	39.60	32.47
**87**	LM48 x LM71	64.25	61.00	109.25	98.00	89.50	79.65	10.81	10.81	10.29	8.60	19.38	16.50	56.13	33.67	37.01	28.32
**88**	LM75 x LM26	56.25	58.25	105.25	99.75	89.48	74.53	10.81	7.88	9.41	7.83	18.88	16.25	42.08	29.13	43.44	28.29
**89**	BW141 x BW162	56.75	56.25	102.50	98.25	85.73	69.03	9.38	12.31	9.75	7.85	18.38	15.00	40.63	26.83	43.12	31.56
**90**	LM71	62.00	61.75	105.50	94.75	73.38	77.50	10.17	7.31	9.75	8.20	17.25	15.13	27.00	27.00	34.88	33.81

DTH = days to 50% heading, DTM = days to 50% maturity, PH = plant height (cm), TN = tiller number, SL = spike length (cm), SPS = spikelets per spike, KPS = kernels per spike, TKW = thousand kernel weight (g), NS = non-stressed, DS = drought-stressed

Table S1 continued.

No.	Genotype	DTH		DTM		PH		TN		SL		SPS		KPS		TKW	
		NS	DS	NS	DS	NS	DS	NS	DS	NS	DS	NS	DS	NS	DS	NS	DS
**91**	LM48	63.00	62.00	104.00	96.00	82.40	67.25	7.88	7.13	8.00	7.60	15.25	13.88	38.63	30.00	31.09	26.75
**92**	BW141 x BW140	53.00	55.50	102.00	100.50	77.60	66.63	7.34	7.25	7.95	7.38	16.38	14.50	30.63	22.46	42.24	37.88
**93**	BW152 x LM70	60.50	61.25	99.50	94.00	94.23	85.00	10.25	12.06	9.99	9.25	18.13	17.13	40.46	25.21	43.14	30.54
**94**	LM47 x BW162	62.50	64.50	103.75	98.25	87.35	83.18	9.88	9.00	8.50	8.81	16.38	17.63	37.21	28.38	43.33	32.95
**95**	LM71 x LM48	58.25	64.50	105.75	97.75	92.48	87.98	13.56	9.00	9.89	9.50	19.13	18.13	39.79	38.29	37.62	29.97
**96**	BW162 x LM71	61.50	59.00	102.75	98.50	90.45	68.78	14.19	6.69	9.90	7.25	18.38	13.25	38.25	25.42	47.92	38.09
**97**	LM47 x LM71	63.25	63.00	103.75	94.75	93.55	76.50	9.38	7.63	9.50	8.10	18.38	14.63	37.42	29.75	42.37	27.64
**98**	LM47	64.50	65.25	106.25	98.00	90.13	71.33	10.13	6.63	9.73	8.49	17.25	15.63	34.33	24.42	45.13	38.10
**99**	LM75 x LM71	59.50	62.00	106.25	95.50	84.53	84.05	12.63	10.50	9.80	8.92	18.75	19.17	43.92	33.06	37.14	24.17
**100**	BW152	61.50	63.00	103.00	96.25	88.65	60.45	14.68	7.19	9.20	6.95	16.38	15.38	31.46	26.04	42.78	33.62
	**Mean**	59.90	59.44	105.90	98.17	87.27	79.76	10.47	9.42	9.14	8.58	17.53	16.51	38.69	32.45	40.89	33.82
	**LSD**	4.14	4.56	6.13	5.63	13.64	13.49	4.99	5.85	1.22	1.78	2.34	3.46	12.25	11.27	7.00	6.94
	**SEM**	0.35	0.29	0.31	0.30	0.64	0.71	0.23	0.21	0.09	0.08	0.17	0.14	0.58	0.48	0.37	0.39
	**CV**	5.90	4.85	2.93	3.04	7.27	8.85	21.66	21.79	9.78	9.46	9.52	8.69	14.96	14.71	9.10	11.39

DTH = days to 50% heading, DTM = days to 50% maturity, PH = plant height (cm), TN = tiller number, SL = spike length (cm), SPS = spikelets per spike, KPS = kernels per spike, TKW = thousand kernel weight (g), LSD = least significant difference, SEM = standard error of mean, %CV = coefficient of variation, NS = non-stressed, DS = drought-stressed

Table S1 continued.

No.	Genotype	SB		RB		PB		RS		GY	
		NS	DS	NS	DS	NS	DS	NS	DS	NS	DS
**1**	LM75	239.58	297.30	49.96	70.25	682.40	680.32	0.29	0.26	335.78	267.32
**2**	LM26 x BW140	233.25	199.69	27.20	54.92	572.95	536.96	0.17	0.29	267.10	241.33
**3**	BW140 x LM71	170.75	177.42	13.00	32.88	443.28	458.36	0.07	0.18	221.82	215.63
**4**	LM47 x BW152	191.85	301.13	13.29	53.94	417.31	594.91	0.06	0.18	209.51	204.99
**5**	LM70 x BW152	308.13	201.93	15.19	40.57	733.45	479.53	0.06	0.25	350.53	202.60
**6**	LM70 x BW141	158.63	188.03	27.72	30.77	584.77	448.72	0.41	0.15	340.52	196.52
**7**	BW162 x LM75	289.19	252.30	37.16	64.09	764.35	537.48	0.10	0.25	436.16	188.96
**8**	LM75 x BW141	231.36	212.91	33.32	28.82	669.32	457.69	0.15	0.12	345.85	184.58
**9**	BW152 x BW141	147.57	265.18	28.12	65.07	514.11	542.62	0.19	0.33	255.81	181.52
**10**	LM70 x BW162	214.65	173.67	26.08	53.36	468.07	438.48	0.21	0.38	223.02	180.73
**11**	BW141 x LM26	338.36	174.62	42.10	28.91	657.34	289.35	0.14	0.22	313.93	178.91
**12**	LM48 x BW140	369.12	155.91	48.37	32.60	962.54	396.74	0.11	0.21	465.86	177.98
**13**	LM75 x LM47	228.06	288.63	28.09	37.59	612.34	519.75	0.13	0.15	304.43	177.73
**14**	LM70 x LM26	197.57	242.52	15.81	54.35	441.47	515.55	0.08	0.28	194.95	172.00
**15**	LM47 x BW140	211.62	216.17	24.29	54.99	582.29	486.55	0.12	0.26	296.05	171.01
**16**	LM26 x LM71	306.70	231.70	41.22	45.08	640.76	476.35	0.14	0.21	290.47	170.58
**17**	BW141 x LM75	304.99	207.99	19.75	49.06	607.51	455.56	0.08	0.23	285.74	169.67
**18**	BW162 x LM26	240.26	243.97	38.05	54.59	544.58	427.52	0.16	0.22	227.57	166.10
**19**	LM26 x BW141	217.49	147.15	37.95	21.70	662.21	362.72	0.21	0.15	347.67	165.70
**20**	LM71 x LM26	388.93	199.61	41.80	50.42	1013.32	443.01	0.16	0.27	497.95	164.94
**21**	BW152 x LM75	302.57	211.06	44.54	32.87	836.91	435.93	0.13	0.20	418.63	164.10
**22**	LM48 x BW162	284.74	154.69	11.01	44.14	590.89	404.77	0.06	0.40	305.60	164.01
**23**	LM75 x BW152	264.24	200.45	31.95	41.28	768.67	396.66	0.13	0.22	403.83	163.14
**24**	BW152 x LM26	194.58	126.02	23.09	30.36	473.93	346.79	0.14	0.29	219.02	162.75
**25**	BW140 x LM75	327.32	174.01	68.54	30.95	851.62	391.42	0.23	0.16	394.29	159.36
**26**	BW140 x BW152	298.51	203.99	28.78	63.06	501.67	402.42	0.13	0.27	265.31	159.29
**27**	BW162 x BW140	168.03	170.87	16.06	57.73	479.04	417.75	0.10	0.30	252.10	158.99
**28**	LM75 x BW140	262.87	186.13	30.43	47.17	685.52	419.07	0.14	0.23	335.23	158.77
**29**	LM71 x BW140	329.99	204.43	31.23	28.99	699.22	418.77	0.12	0.17	295.56	158.42
**30**	BW141 x LM70	375.67	359.04	22.18	25.14	822.03	569.22	0.08	0.11	362.55	158.15

SB = shoot biomass (g m-2), RB = root biomass (g m-2), PB = total plant biomass (g m-2), RS = root to shoot ratio and GY = grain yield (g m-2), NS = non-stressed, DS = drought-stressed

Table S1 continued.

No.	Genotype	SB		RB		PB		RS		GY	
		NS	DS	NS	DS	NS	DS	NS	DS	NS	DS
**31**	LM75 x LM48	248.28	227.76	24.20	52.55	541.43	464.02	0.12	0.29	282.93	157.01
**32**	BW141 x BW152	354.91	175.07	38.04	34.05	737.05	392.32	0.13	0.22	294.10	156.58
**33**	LM75 x LM70	228.70	172.53	28.18	17.56	528.53	371.14	0.14	0.09	232.17	154.74
**34**	LM70 x LM47	275.18	286.85	28.72	36.80	842.77	484.41	0.09	0.14	374.15	154.45
**35**	LM26 x LM48	234.84	133.14	29.92	32.37	692.11	345.19	0.14	0.27	365.26	153.57
**36**	BW162	307.79	124.96	26.75	28.12	771.99	331.71	0.12	0.28	373.88	152.67
**37**	BW140 x LM70	228.48	143.97	28.62	47.66	540.94	369.63	0.13	0.65	242.60	152.14
**38**	LM70 x LM75	371.09	265.52	47.30	29.55	947.47	472.59	0.13	0.10	452.21	151.73
**39**	LM47 x LM70	181.44	246.43	24.38	38.46	412.26	461.89	0.14	0.16	176.44	151.28
**40**	LM47 x LM48	248.33	251.02	25.92	27.09	586.48	480.80	0.12	0.12	266.87	151.00
**41**	LM26 x BW162	264.61	272.42	34.58	50.50	737.26	498.24	0.14	0.19	374.41	149.85
**42**	LM48 x LM26	223.14	231.05	26.10	49.37	673.73	454.77	0.12	0.22	362.82	149.02
**43**	BW162 x LM48	270.60	218.48	34.92	56.46	647.42	446.25	0.13	0.29	292.22	146.42
**44**	BW141	167.72	164.62	15.23	23.32	411.06	358.36	0.10	0.16	194.97	145.66
**45**	LM75 x BW162	300.98	176.51	33.09	30.00	701.57	375.95	0.11	0.19	314.11	144.82
**46**	BW140 x BW162	244.74	115.98	38.52	16.51	667.47	299.28	0.19	0.20	328.38	142.56
**47**	LM71 x LM75	413.78	184.35	43.39	38.42	1019.04	389.43	0.16	0.27	480.24	142.45
**48**	LM70 x BW140	156.74	149.50	45.10	17.02	435.10	332.62	0.66	0.13	197.30	141.96
**49**	LM26 x LM75	350.90	211.09	40.12	53.31	954.03	429.96	0.15	0.28	481.21	141.50
**50**	BW162 x BW141	266.05	180.52	75.71	43.27	776.54	386.17	0.26	0.23	371.60	138.78
**51**	BW140 x LM48	112.54	125.04	7.14	23.04	278.22	308.84	0.07	0.22	151.68	137.40
**52**	LM26	291.13	122.65	17.21	28.51	674.12	276.83	0.07	0.60	312.63	137.19
**53**	LM48 x BW141	287.87	156.44	39.73	24.30	602.09	340.23	0.20	0.21	234.61	136.32
**54**	LM47 x LM26	282.00	192.87	51.35	28.92	804.19	380.20	0.17	0.16	449.20	135.81
**55**	BW140 x BW141	167.00	121.89	20.39	20.09	460.77	300.00	0.11	0.24	238.01	135.06
**56**	BW152 x LM48	267.76	178.29	22.87	38.54	692.08	374.66	0.09	0.16	343.12	134.90
**57**	BW140	281.02	169.50	27.62	42.47	656.31	365.69	0.09	0.27	353.33	131.38
**58**	LM70 x LM71	186.01	289.16	22.67	64.86	522.58	507.64	0.18	0.24	266.99	131.30
**59**	LM26 x LM47	322.08	166.32	37.34	36.65	768.22	356.54	0.12	0.28	349.40	131.25
**60**	BW162 x BW152	242.65	244.84	53.62	32.65	644.57	455.70	0.23	0.21	297.69	130.49

SB = shoot biomass (g m-2), RB = root biomass (g m-2), PB = total plant biomass (g m-2), RS = root to shoot ratio and GY = grain yield (g m-2), NS = non-stressed, DS = drought-stressed

Table S1 continued.

No.	Genotype	SB		RB		PB		RS		GY	
		NS	DS	NS	DS	NS	DS	NS	DS	NS	DS
**61**	LM71 x BW152	225.04	163.97	21.24	34.45	604.82	359.81	0.12	0.22	227.61	130.41
**62**	LM48 x LM47	299.65	120.72	26.80	30.80	552.61	303.67	0.10	0.34	235.58	130.04
**63**	BW140 x LM26	283.59	150.38	31.09	24.11	670.11	325.65	0.11	0.19	303.78	129.20
**64**	LM47 x LM75	254.44	212.38	33.94	31.35	634.92	393.28	0.13	0.16	296.19	127.82
**65**	LM26 x LM70	369.95	197.27	31.99	17.41	625.15	395.97	0.08	0.20	258.98	127.72
**66**	LM71 x LM70	138.22	146.78	18.28	41.87	411.76	337.39	0.15	0.26	233.06	127.13
**67**	LM48 x LM75	275.22	123.82	25.69	21.65	765.71	237.56	0.14	0.24	397.27	125.23
**68**	LM48 x LM70	337.22	181.24	18.41	59.07	800.17	385.80	0.08	0.30	379.95	124.35
**69**	LM48 x BW152	174.58	200.71	50.77	28.29	546.33	346.80	0.25	0.23	274.34	124.07
**70**	BW140 x LM47	184.46	230.98	46.98	69.38	556.07	444.20	0.46	0.29	277.45	122.94
**71**	LM71 x BW141	162.61	160.60	23.11	45.84	478.25	349.70	0.27	0.26	250.02	122.44
**72**	BW141 x LM71	173.86	96.06	20.55	18.39	427.39	231.54	0.13	0.14	199.13	119.20
**73**	LM70	365.06	245.33	19.11	42.40	847.96	426.63	0.07	0.17	396.39	118.71
**74**	BW152 x BW140	288.63	160.45	23.31	53.34	730.11	393.69	0.08	0.31	357.41	118.60
**75**	BW152 x BW162	170.07	131.74	18.00	17.57	450.42	272.57	0.10	0.22	224.23	117.65
**76**	LM26 x BW152	348.96	157.93	44.96	27.50	910.06	322.86	0.21	0.17	441.14	117.46
**77**	BW141 x LM48	229.43	119.85	23.30	18.31	683.91	280.77	0.11	0.25	356.58	117.25
**78**	LM70 x LM48	180.83	128.33	10.03	12.15	516.84	287.19	0.10	0.17	271.77	116.67
**79**	LM47 x BW141	253.48	179.62	31.28	73.29	595.31	387.78	0.12	0.44	265.43	115.28
**80**	LM71 x LM47	244.24	167.27	26.54	28.76	545.48	284.85	0.13	0.37	276.88	115.09
**81**	BW141 x LM47	236.86	104.70	32.00	25.11	598.02	262.04	0.18	0.41	281.34	113.02
**82**	BW162 x LM70	102.23	98.74	13.59	39.68	270.87	265.10	0.13	0.42	176.69	112.73
**83**	BW162 x LM47	226.81	205.60	33.90	39.29	595.36	374.49	0.21	0.19	292.67	110.77
**84**	BW152 x LM71	151.59	189.16	42.60	44.03	562.41	357.08	0.24	0.20	226.02	105.89
**85**	LM71 x BW162	374.76	162.53	31.60	30.87	830.54	296.63	0.09	0.30	362.54	104.44
**86**	BW152 x LM47	269.27	148.63	23.75	36.37	585.16	306.76	0.09	0.25	249.69	104.06
**87**	LM48 x LM71	326.32	221.77	36.43	82.97	897.12	424.90	0.19	0.38	456.72	102.69
**88**	LM75 x LM26	274.35	102.73	26.17	30.51	764.08	252.22	0.10	0.33	396.20	101.69
**89**	BW141 x BW162	186.70	153.22	25.53	33.58	525.70	304.95	0.17	0.24	267.93	100.99
**90**	LM71	216.93	130.79	36.02	28.57	593.20	277.39	0.14	0.20	241.39	100.87

SB = shoot biomass (g m-2), RB = root biomass (g m-2), PB = total plant biomass (g m-2), RS = root to shoot ratio and GY = grain yield (g m-2), NS = non-stressed, DS = drought-stressed

Table S1 continued.

No.	Genotype	SB		RB		PB		RS		GY	
		NS	DS	NS	DS	NS	DS	NS	DS	NS	DS
**91**	LM48	153.67	106.63	13.73	21.78	434.85	208.83	0.10	0.41	228.59	100.27
**92**	BW141 x BW140	165.03	125.72	24.59	27.37	462.64	270.09	0.15	0.34	233.34	100.01
**93**	BW152 x LM70	267.00	272.00	54.38	28.83	672.66	360.14	0.19	0.11	300.24	99.55
**94**	LM47 x BW162	204.81	176.25	41.11	36.82	510.12	329.31	0.23	0.21	225.81	99.36
**95**	LM71 x LM48	321.24	196.36	58.69	40.68	824.41	353.02	0.18	0.32	379.90	99.13
**96**	BW162 x LM71	334.12	103.75	25.92	14.65	809.10	225.54	0.09	0.18	432.71	91.57
**97**	LM47 x LM71	277.87	196.21	27.94	31.15	715.38	283.55	0.12	0.20	350.05	89.96
**98**	LM47	271.05	151.17	22.85	32.64	657.22	287.85	0.08	0.20	310.53	88.92
**99**	LM75 x LM71	323.36	347.87	46.38	73.42	763.69	505.65	0.15	0.21	336.71	72.11
**100**	BW152	399.15	121.55	31.00	23.24	922.43	199.76	0.09	0.32	420.74	67.00
	**Mean**	256.20	186.60	30.99	38.06	643.60	384.80	0.15	0.24	310.10	141.20
	**LSD**	141.90	142.30	31.26	33.76	342.70	250.60	0.23	0.23	183.10	95.90
	**SEM**	6.97	5.52	1.24	1.51	15.71	9.04	0.01	0.01	7.87	3.37
	**CV**	27.21	29.59	40.07	39.55	24.41	23.50	56.06	38.27	25.37	23.87

SB = shoot biomass (g m^{-2}), RB = root biomass (g m^{-2}), PB = total plant biomass (g m^{-2}), RS = root to shoot ratio and GY = grain yield (g m^{-2}), LSD = least significant difference, SEM = standard error of mean, %CV = coefficient of variation, NS = non-stressed, DS = drought-stressed

Table S2. Direct (bold face values) and indirect effects for yield components and root attributes on grain yield of 10 bread wheat parental lines and 90 F_2 progenies under drought-stressed conditions at two sites.

Traits	DTH	DTM	PH	TN	SL	SPS	KPS	TKW	SB	RB	PB	RS	GY
**DTH**	**-0.19**	0.06	-0.05	0.01	-0.05	-0.02	0.01	0.03	-0.03	-0.01	0.01	-0.01	-0.38***
**DTM**	-0.02	**0.06**	-0.02	-0.01	-0.02	-0.01	0.00	0.02	-0.01	0.00	0.00	0.01	0.24*
**PH**	0.01	-0.01	**0.03**	0.01	0.03	0.02	0.01	-0.01	0.02	0.01	0.02	-0.01	0.26**
**TN**	0.00	0.00	0.01	**0.03**	0.01	0.01	0.01	-0.01	0.02	0.02	0.02	0.00	0.34***
**SL**	0.00	0.00	0.00	0.00	**0.00**	0.00	0.00	0.00	0.00	0.00	0.00	0.00	0.24*
**SPS**	-0.02	0.02	-0.08	-0.04	-0.10	**-0.13**	-0.07	0.05	-0.07	-0.05	-0.07	0.02	0.26*
**KPS**	-0.01	-0.02	0.13	0.08	0.17	0.18	**0.34**	-0.10	0.14	0.08	0.17	-0.04	0.55***
**TKW**	-0.02	0.06	-0.03	-0.04	-0.04	-0.06	-0.05	**0.16**	-0.07	-0.06	-0.04	-0.01	0.09
**SB**	-0.06	0.09	-0.29	-0.22	-0.26	-0.24	-0.17	0.16	**-0.41**	-0.24	-0.36	0.13	0.39***
**RB**	-0.01	0.01	-0.06	-0.08	-0.06	-0.06	-0.04	0.05	-0.09	**-0.15**	-0.10	-0.06	0.28**
**PB**	-0.06	-0.03	0.60	0.57	0.54	0.55	0.50	-0.26	0.88	0.65	**1.00**	-0.19	0.68***
**RS**	0.00	0.00	-0.01	0.00	0.00	0.00	0.00	0.00	-0.01	0.01	-0.01	**0.03**	-0.14

* significant at $P < 0.05$; ** $P < 0.01$, DTH = days to 50% heading, DTM = days to 50% maturity, PH = plant height (cm), TN = tiller number, SL = spike length (cm), SPS = spikelets per spike, KPS = kernels per spike, TKW = thousand kernel weight (g), SB = shoot biomass (g m^{-2}), RB = root biomass (g m^{-2}), PB = total plant biomass (g m^{-2}), RS = root to shoot ratio and GY = grain yield (g m^{-2}).

Table S3. Direct (bold face values) and indirect effects for yield components and root attributes on grain yield of 10 bread wheat parental lines and 90 F_2 progenies under non-stressed conditions at two sites.

Traits	DTH	DTM	PH	TN	SL	SPS	KPS	TKW	SB	RB	PB	RS	GY
**DTH**	**-0.02**	0.00	-0.01	0.00	0.00	0.00	0.00	0.00	0.00	0.00	0.00	0.00	0.02
**DTM**	0.00	**0.03**	0.00	0.00	0.00	0.01	0.00	-0.01	0.00	0.00	0.00	0.00	0.02
**PH**	0.00	0.00	**0.00**	0.00	0.00	0.00	0.00	0.00	0.00	0.00	0.00	0.00	0.48***
**TN**	0.00	0.00	0.03	**0.08**	0.02	0.02	0.01	0.00	0.05	0.03	0.05	0.00	0.60***
**SL**	0.00	0.00	0.00	0.00	**-0.01**	-0.01	0.00	0.00	0.00	0.00	0.00	0.00	0.41***
**SPS**	0.00	0.00	0.01	0.00	0.01	**0.01**	0.01	0.00	0.01	0.00	0.01	0.00	0.48***
**KPS**	0.02	0.01	0.04	0.02	0.06	0.08	**0.13**	-0.03	0.04	0.01	0.05	-0.02	0.48***
**TKW**	0.00	-0.02	0.02	0.00	0.01	0.00	-0.02	**0.08**	0.00	0.01	0.01	0.01	0.13
**SB**	-0.02	-0.02	-0.10	-0.11	-0.09	-0.09	-0.06	-0.01	**-0.19**	-0.07	-0.16	0.05	0.73***
**RB**	0.02	0.00	-0.04	-0.06	-0.03	-0.03	-0.01	-0.01	-0.05	**-0.13**	-0.07	-0.09	0.41***
**PB**	0.04	0.03	0.55	0.66	0.44	0.50	0.43	0.11	0.89	0.52	**1.06**	-0.05	0.94***
**RS**	-0.01	0.00	0.00	0.00	0.00	-0.01	-0.01	0.01	-0.02	0.04	0.00	**0.07**	-0.03

* significant at $P < 0.05$; ** $P < 0.01$, DTH = days to 50% heading, DTM = days to 50% maturity, PH = plant height (cm), TN = tiller number, SL = spike length (cm), SPS = spikelets per spike, KPS = kernels per spike, TKW = thousand kernel weight (g), SB = shoot biomass (g m^{-2}), RB = root biomass (g m^{-2}), PB = total plant biomass (g m^{-2}), RS = root to shoot ratio and GY = grain yield (g m^{-2}).
